# Temperature Effect on the Stability of the Polarized State Created by Local Electric Fields in Strontium Barium Niobate Single Crystals

**DOI:** 10.1038/s41598-017-00172-1

**Published:** 2017-03-09

**Authors:** V. Ya. Shur, V. A. Shikhova, D. O. Alikin, V. A. Lebedev, L. I. Ivleva, J. Dec, D. C. Lupascu, V. V. Shvartsman

**Affiliations:** 1 School of Natural Sciences and Mathematics, Ural Federal University, 620000 Ekaterinburg, 51 Lenin Ave., Russia; 2 Faculty of Materials Science, Lomonosov Moscow State University, 119991 Moscow, GSP-1, 1-73 Leninskiye Gory, Russia; 3 Prokhorov General Physics Institute, Russian Academy of Sciences, 119991 Moscow, 38 Vavilova str., Russia; 4 Institute of Materials Science, University of Silesia, 40-007 Katowice, 4 Uniwersytecka str., Poland; 5 Institute for Materials Science and Center for Nanointegration Duisburg-Essen (CENIDE), University of Duisburg-Essen, 45141 Essen, Universitätsstraße15, Germany

## Abstract

The stability of ferroelectric domain patterns at the nanoscale has been a topic of much interest for many years. We investigated the relaxation of the polarized state created by application of a local electric field using a conductive tip of a scanning probe microscope for the model uniaxial relaxor system Sr_x_Ba_1−x_Nb_2_O_6_ (SBN) in its pure and Ce-doped form. The temporal relaxation of the induced PFM contrast was measured at various temperatures. The average value of the induced contrast decreases during heating for all investigated crystals. Below the freezing temperature the induced state remains stable after an initial relaxation. Above the freezing temperature the induced state is unstable and gradually decays with time. The stability of the induced state is strongly affected by the measuring conditions, so continuous scanning results in a faster decay of the poled domain. The obtained effects are attributed to a decrease of the induced polarization and backswitching of the polarized area under the action of the depolarization field.

## Introduction

The creation of regular micron and submicron domain patterns by application of a local electric field using a conductive tip of a scanning probe microscope (SPM)^1^ and their stability have been intensively studied due to possible applications in optoelectronics, nonlinear optics, and piezoelectricity. Especially light frequency conversion devices based on periodically poled ferroelectric crystals^2^ and high-density storage systems^3^ are very promising. Nanoscopic studies of the domain structure evolution under application of a local field by a conductive SPM tip provide useful insight into the polarization reversal process in ferroelectrics. The majority of these studies are concerned with thin crystals of LiNbO_3_ and LiTaO_3_^4–6^ using high-voltage SPM^7^ due to the extremely high coercive fields of these materials.

The uniaxial relaxor ferroelectric strontium barium niobate (Sr_*x*_Ba_1−*x*_Nb_2_O_6_, SBN100*x*) is considered to be a prospective material for domain engineering and high-density storage systems. Prominent nonlinear-optical and electro-optical properties of SBN together with high values of piezoelectric coefficients^8^ open new horizons for commercialization of the domain patterned crystals. Furthermore, SBN crystals are very attractive for SPM recording due to low coercive fields^8^. However, it is considered that the intrinsic nanoscale compositional inhomogeneity in relaxor ferroelectrics hampers the creation of tailored domain structures in SBN^9^.

The first high-resolution studies using SPM allowed visualizing pristine static domain patterns on the polar surface of SBN crystals^10–12^. In particular, piezoresponse force microscopy (PFM) revealed maze-type domain structures with characteristic sizes of about a hundred nanometers^11^ in thermally depolarized crystals at room temperature, as well as its evolution at elevated temperatures^12^. The micro- and nanodomain structures formed during polarization reversal in a uniform field were also studied using PFM^13^.

The field and exposure characteristics of the SPM domain recording were investigated in SBN61 crystals^14^. An important issue is the stability of the created domains, especially considering possible applications. It was revealed that 1D and 2D regular domain arrays recorded on polar surfaces at room temperature are essentially more stable as compared to isolated domains^14^. The fabrication of domain gratings with charged domain walls on the (100) nonpolar surface (*X*-cut) of SBN61 at room temperature and its temporal relaxation also have been demonstrated^15^.

Most of the studies have addressed the stability of the domain patterns created by SPM at room temperature. The effect of temperature has been less investigated^10^. Due to the relatively low transition temperature SBN single crystals are good candidates for SPM investigation of induced domain states both in the relaxor state (above the freezing temperature) as well as in the ferroelectric state (below the freezing temperature).

SBN crystals belong to a class of relaxor ferroelectrics^16^. These materials have a particular polar structure characterized by appearance of so-called polar nanoregions, PNRs, at relatively high temperatures (~600 K for SBN75^17^). PNRs have size of a few nanometers. This relaxor state differs from the paraelectric one, but manifests an ergodic behaviour^18^. In particular, by application of a strong enough electrical field a macroscopically poled state can be induced, which is however unstable and reverses back to the relaxor state after the field is switched off. Strengthening of interaction between PNRs on cooling results in a transition into a non-ergodic superdipolar glass state characterized by transformation of PNRs in coarse-grained microdomains that keep nanometer scale size^19, 20^. This occurs at so-called freezing temperature. When relaxor ferroelectrics are poled below the freezing temperature an irreversible transition into a ferroelectric state can be induced.

In this paper, we report on a study of PFM-recorded states in SBN crystals with different transitions temperatures: SBN75 with the transition below room temperature and SBN61 (pure and Ce-doped) with the transition above room temperature. The effect of temperature on both the induced piezoresponse and the stability of the created domains was investigated. Full and a partial relaxation of the induced state was observed above and below the transition temperature, respectively. Based on obtained data we have concluded that decay of the domains is promoted by the incomplete screened depolarization field. We also found that the measuring conditions affect the stability of domains. Continuous scanning removes screening charges and accelerates domains backswitching.

## Results

The experiments were done for the multi-domain state that was produced by thermal depolarization achieved by heating up the sample to 200 °C and subsequent zero-field cooling.

**Figure 1:**
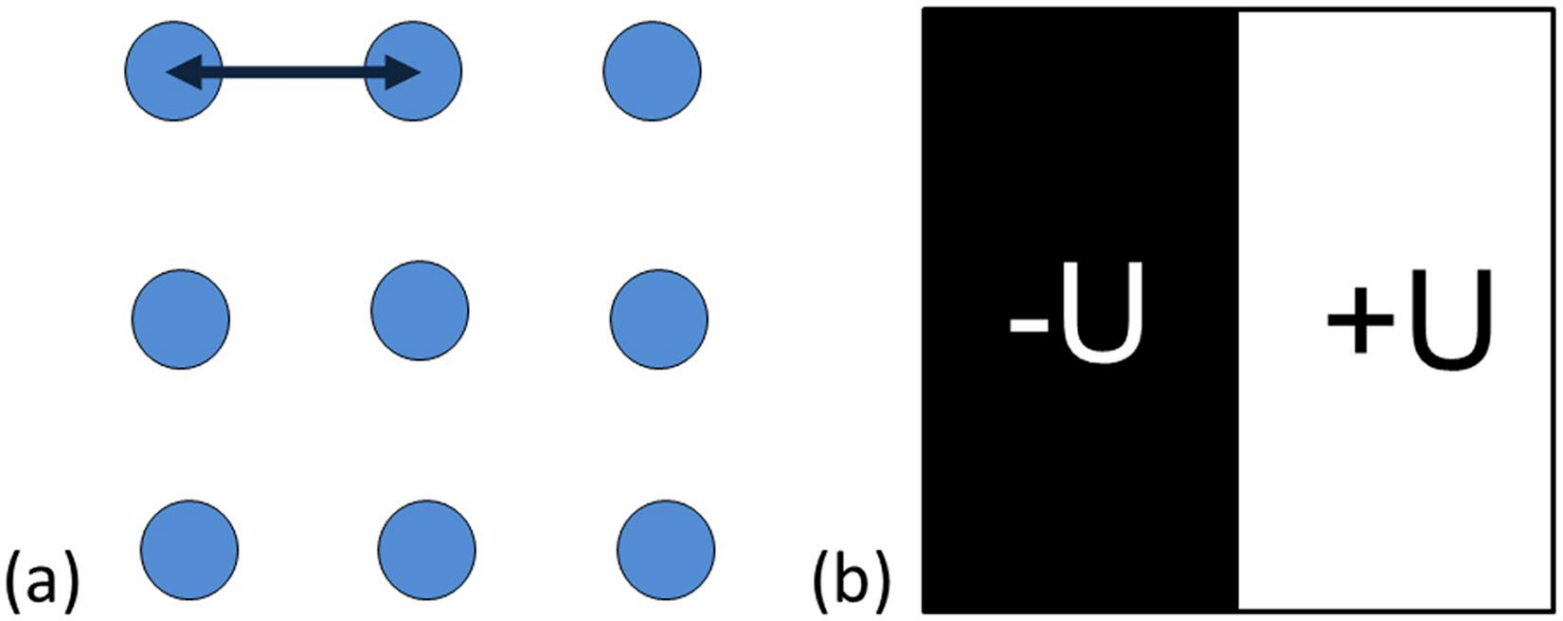
Schemes of creation of polarized states by (**a**) “discrete” and (**b**) “continuous” scanning.

The polarized states were created by the application of a local electric field using a conductive SPM tip in regimes of either “discrete” or “continuous” scanning. The discrete scanning scheme is as follows: *dc* voltage pulses with magnitude 100 V were applied for 1 or 10 s over a spatial grid of 3 × 3 or 5 × 5 points with 5 μm distance between the points (Fig. 1a). For “continuous” scanning a *dc* voltage of 40–50 V was applied over a matrix of 256 × 256 dots with a recording time of approximately 1 ms/dot, and a distance between the dots of 20 nm (Fig. 1b). The polarity of the applied voltage was reversed to the opposite one for half of the scanned area (Fig. 1b).

**Figure 2:**
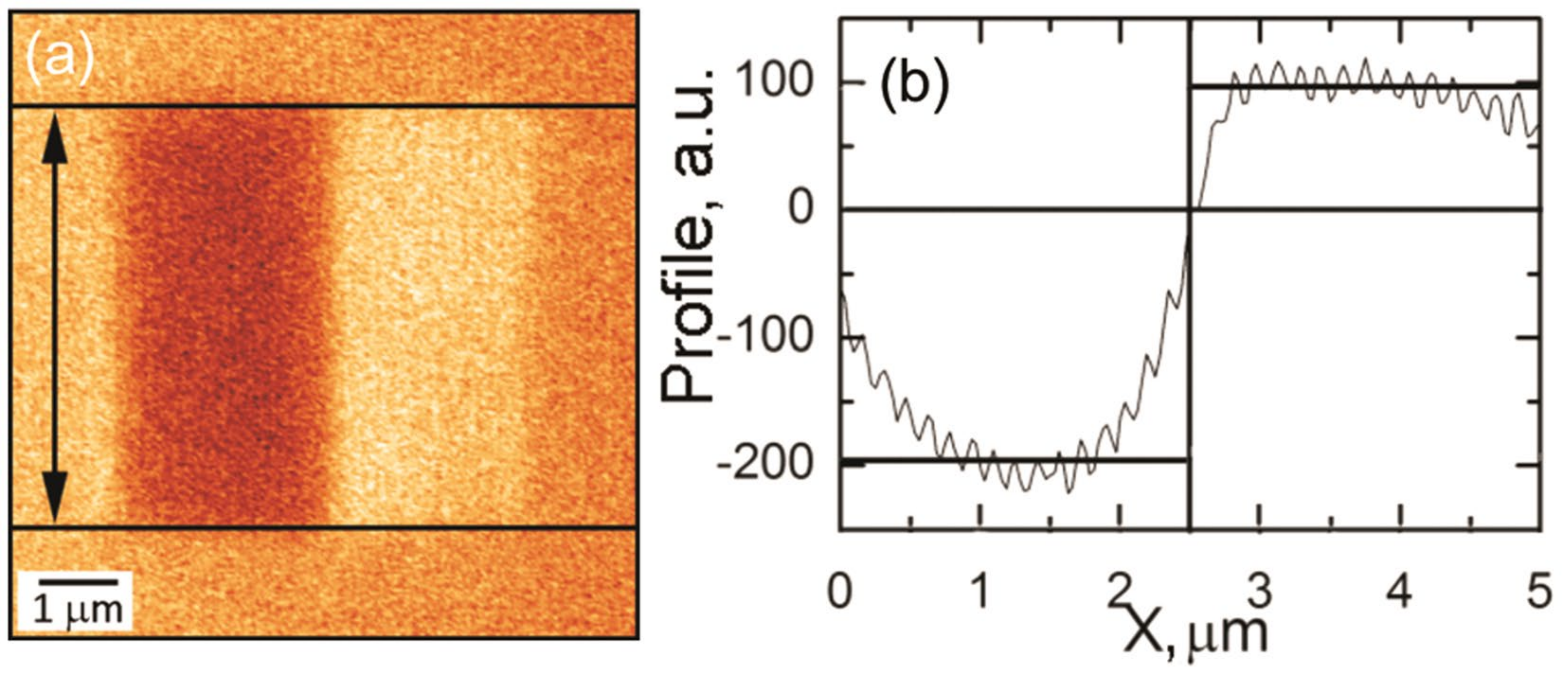
(**a**) Typical PFM image of the polarized area recorded by “continuous” scanning and (**b**) averaged PFM signal across the poled region.

The piezoelectric response was characterized by the value of the averaged PFM contrast, difference of piezoelectric response between the polarized areas produced by application of fields of opposite sign. To estimate it and increase signal/noise ratio we averaged over all cross-sections of the PFM image inside the poled region (Fig. 2). The time and temperature dependences of the normalized PFM contrast (relative piezoresponse) were analyzed. For domains created by discrete scanning the effective diameter and the average piezoelectric response were analyzed for different poling voltages and temperatures.

### **“**Continuous” scanning

#### SBN75

**Figure 3:**
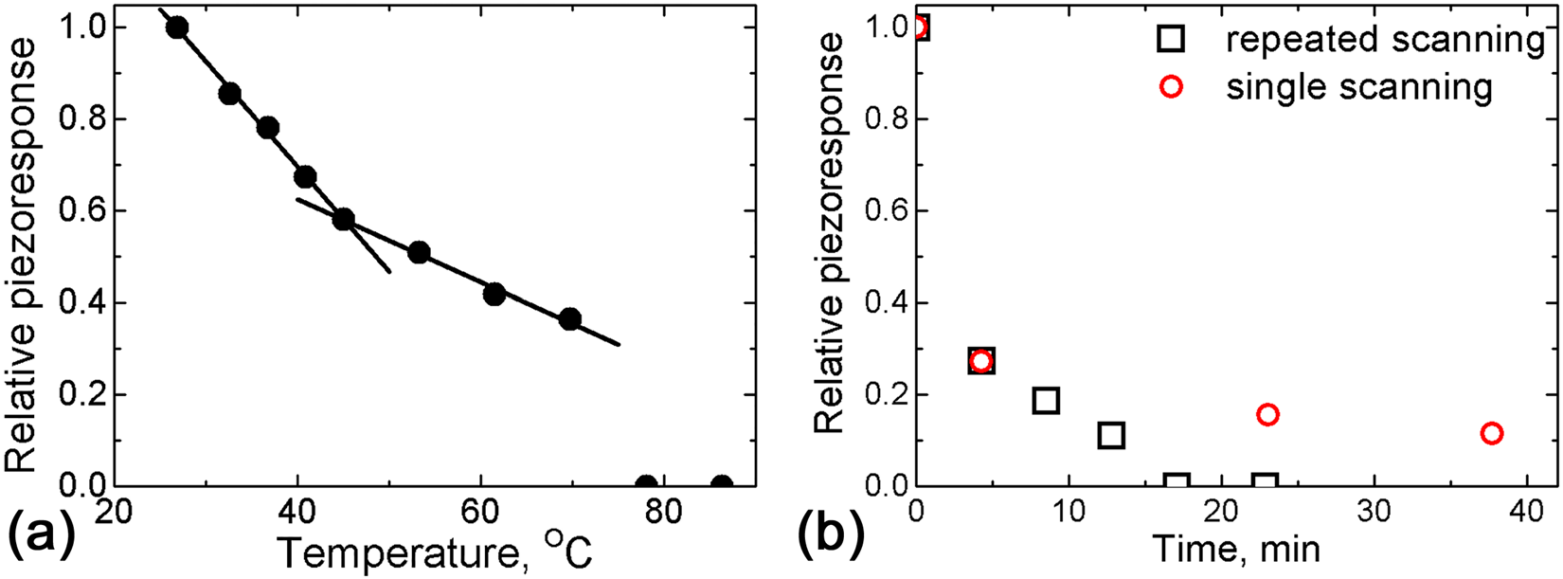
(**a**) Temperature dependence of the relative piezoresponse of the polarized areas in SBN75. Experimental points are fitted by linear dependences. (**b**) Time dependence of the relative PFM contrast of the polarized areas in SBN75 for single and repeated scanning at room temperature. The poling voltage *U*_*dc*_ = 40 V.

First, we present relaxation of the PFM contrast inside the polarized areas created at various temperatures in thermally depoled SBN75. The temperature dependence of the relative piezoresponse is shown in Fig. 3a. The data correspond to the first scan (~4 minutes) after the poling procedure. They are normalized to the value of the average piezoresponse at room temperature. One can see that the PFM contrast decreases with increasing temperature and vanishes above 70 °C. The slope of the temperature dependence of the PFM contrast changes by 2.5 times near 45 °C (Fig. 3a). At approximately the same temperature the macroscopic polarization hysteresis loops disappear in this sample^21^.

It is significant that the created domains in SBN75 are unstable, and isothermal decay of the PFM contrast is observed at all studied temperatures. We found that the rate of this relaxation process essentially depends on the scanning conditions. For continuous scanning at room temperature, the created contrast disappears completely already after four scanning cycles (17 min). Whereas when the tip is elevated after finishing the first scan, the contrast is preserved even after 38 min (Fig. 3b). This fact clearly evidences that the scanning process itself essentially influences the stability of the induced poled state.

### SBN61

**Figure 4:**
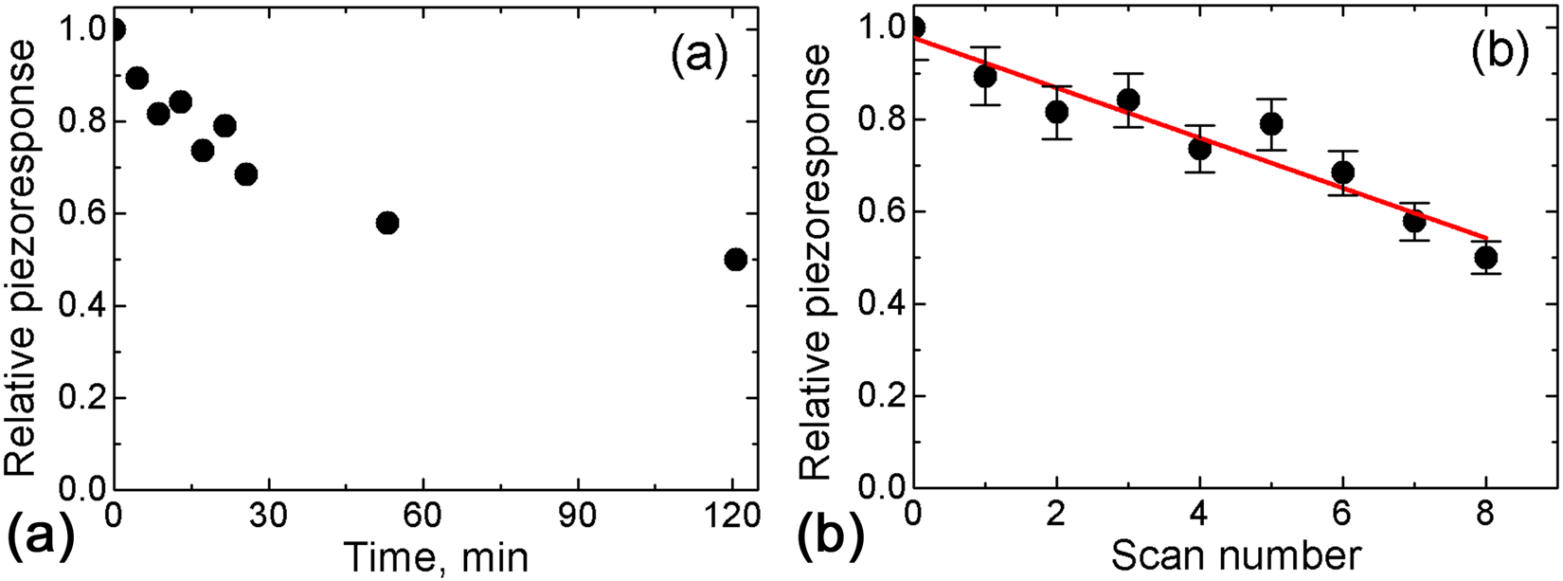
(**a**) Time and (**b**) scan number dependences of the relative PFM contrast for repeated scanning at room temperature after creation of polarized areas in SBN61. (**b**) Experimental points are fitted by a linear dependence. The poling voltage is *U*_*dc*_ = 50 V.

For SBN61 at room temperature the induced polarization was essentially more stable than in SBN75. The relative piezoreponse for the polarized areas created at room temperature decreased with time to half of the initial value after 120 min (Fig. 4a). We found that it scales linearly with the number of consecutive scans (Fig. 4b).

**Figure 5:**
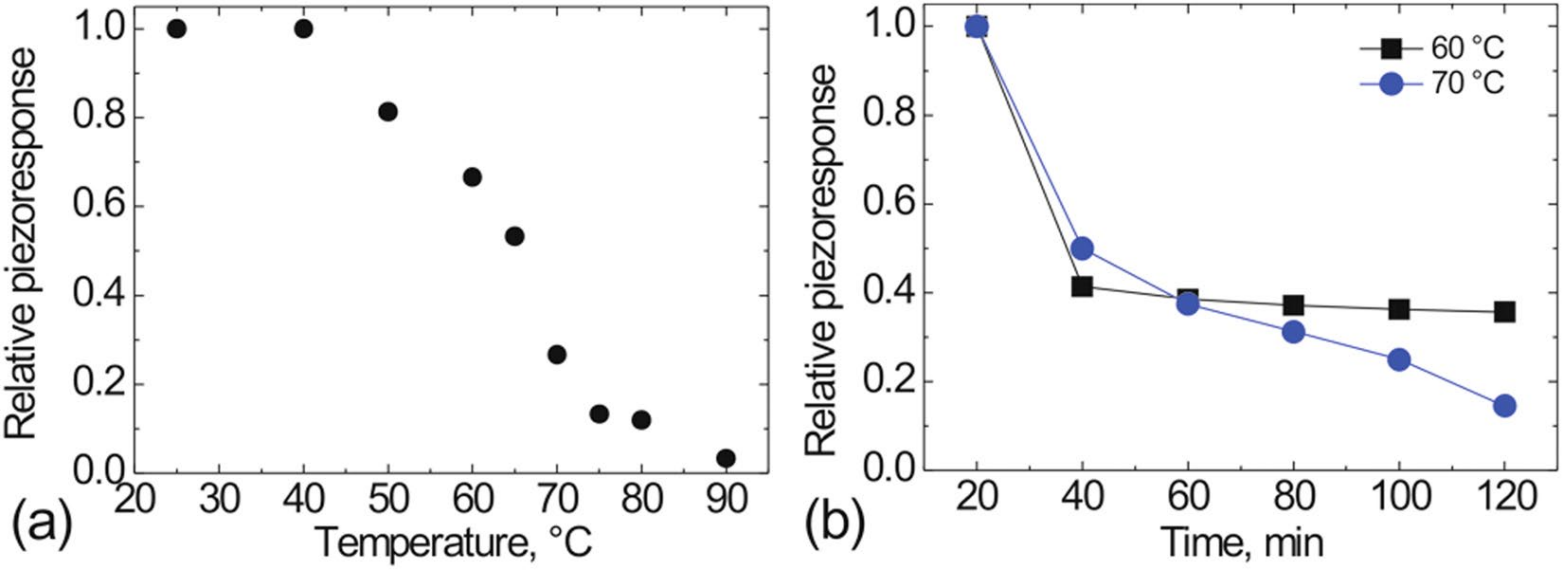
(**a**) The temperature dependence of the relative PFM contrast for repeated scanning during heating after creation of polarized areas in SBN61 at room temperature, *U*_*dc*_ = 40 V. (**b**) Time dependence of the induced piezoresponse below and above the freezing temperature.

Figure 5a shows the temperature relaxation of the relative piezoresponse in polarized areas created in SBN61 at room temperature measured by repeated scanning during heating up to 90 °C with an average heating rate of about 0.3 °C/min. A strong decay of the piezoresponse is observed between 60 °C and 70 °C, i.e. close to the freezing temperature (*T*_*f*_ = 65 °C) for this composition. To compare the stability of the induced state below and above the freezing temperature we analyzed time dependences of the induced piezoresponse at 60 °C and 70 °C (Fig. 5b). In the first case, after a strong initial decay the piezoresponse remained stable at about 40% of the initial value. For 70 °C, however, the piezoresponse decreased continuously to zero, similar to the observation for SBN75.

### **“**Discrete” scanning

**Figure 6:**
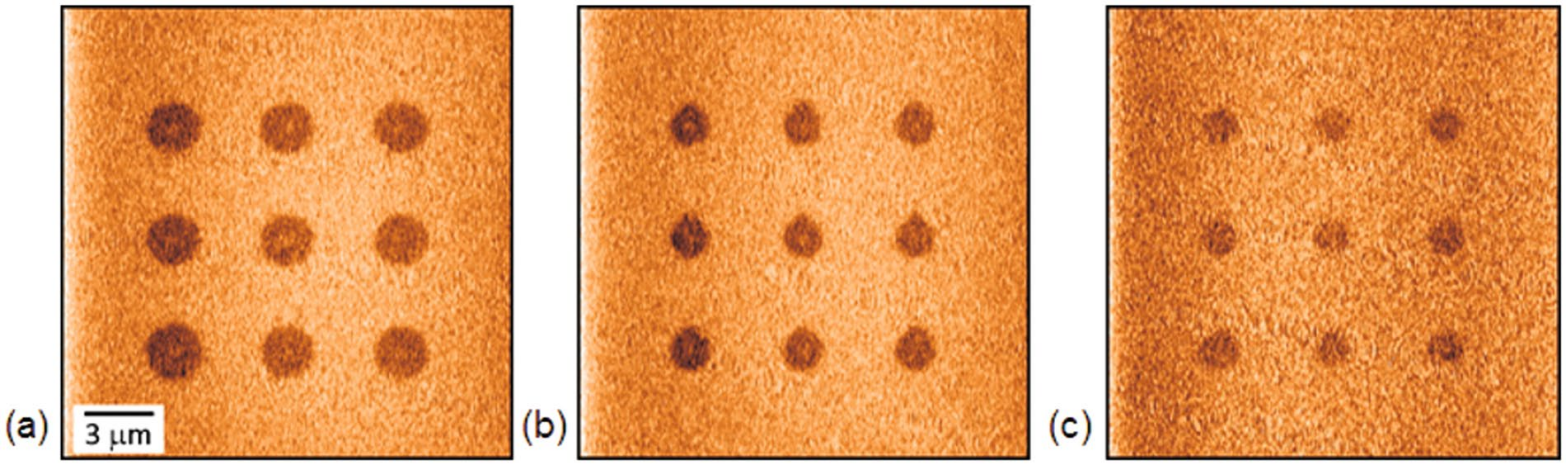
The PFM images of polarized regions created by discrete scanning in SBN61:Ce at various temperatures: (**a**) 23 °C, (**b**) 39 °C, (**c**) 55 °C. *U*_*dc*_ = 100 V, pulse duration 10 s.

**Figure 7:**
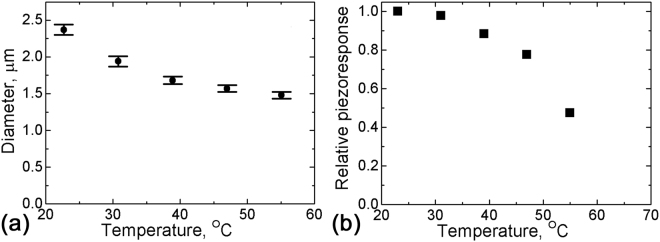
The temperature dependences of (**a**) the average diameter and (**b**) the relative PFM contrast of the circular domains created by discrete scanning in SBN61:Ce. *U*_*dc*_ = 100 V, pulse duration 10 s.

The temperature dependence of the sizes of isolated domains created by discrete scanning was studied in a SBN61:Ce single crystal (*U*_*dc*_ = 100 V, pulse duration 10 s, the distance between domain centers is 5 μm) (Fig. 6). The average diameter of the created circular shaped domains and their relative PFM contrast decrease with increasing temperature (Fig. 7).

## Discussion

We observed that in both the continuous and the discrete regime the induced domains exhibit at least a partial backswitching after removing the external field. Besides, there is a qualitative difference between experiments performed below and above the freezing temperature. For temperatures corresponding to the relaxor phase (that is above the freezing temperature) the sample comes back to the unpoled state after a certain time. The domains written below the freezing temperature are more stable. After initial gradual backswitching both the induced piezoresponse and the diameter of the created domain (in the case of discrete switching) approaches a steady value.

The measured PFM signal is dominated by the electromechanical contribution due to the converse piezoelectric effect. Since the piezoelectric coefficient is proportional to the value of spontaneous polarization, the variation of the PFM signal reflects the variation of the polarization. In spite of the relatively “hard” cantilevers (the spring constant lies in range 15–50 N/m) were used, the electrostatic contribution could not be eliminated completely. It is proportional to the dielectric permittivity of the sample and the work function difference between the tip and sample surface. This electrostatic signal is unipolar and results in some asymmetry of the measured PFM signal relative to zero value (see e.g. Fig. 2b). However it does not affect the analyzed PFM contrast, the difference between piezoresponse in positively and negatively poled areas.

The relaxation of the PFM contrast created by field application reflects the relaxation of the averaged polarization. This can be attributed to two mechanisms: 1) spontaneous decay of the created domain structure (backswitching), and/or 2) decrease of the magnitude of induced/spontaneous polarization.

The backswitching effect represents the slow partial recovery of the initial multi domain state under the action of the residual (incompletely screened) depolarization field^22^. In PFM in absence of a macroscopic electrode, the depolarization field is screened by both surface charges (external screening) and bulk charges (intrinsic screening).

The studied samples with thickness from 0.2 to 0.5 mm are rather thick in PFM sense. This means that under the applied poling voltage the created domains never reach the opposite polar surface (bottom electrode)^23^. The resulting domain wall will be charged to screen the arising depolarization field (intrinsic screening). It is known that such a domain wall may attain a particular zig-zag like profile to minimize the depolarizing field^24, 25^. After the electric field is switched off the domain wall may start to move back towards the free sample surface, but may be also pinned by bulk screening charges. This relaxation can contribute to the observed isothermal decay of the piezoresponse, i.e. backswitching. However, separation of this contribution as well as information about structure of this domain wall cannot be obtained from the presented PFM data. This should be matter of a separate study.

We have observed experimentally that the depolarization effect is accelerated for continuous scanning, namely the piezoresponse contrast linearly decays with the number of full scans (Figs 3 and 4). This is in accordance with reports that the grounded PFM tip in contact with the created domains promotes its backswitching^26, 27^. We assume that during scanning the tip plays the role of a movable ground electrode and removes the screening surface charges. This effectively enhances the depolarization field and leads to destabilization of the created domains and accelerated backswitching. This observation indicates that the surface screening conditions play significant role in the compensation of the depolarization field.

We have also observed that the induced polarization became less stable at higher temperature (Figs 3a and 5). The decay of the created domain structures is typically spatially inhomogeneous (Supplementary Information Fig. S1). On the one hand, the thermally activated charge carrier diffusion becomes stronger with increasing temperature, which should result in the dissipation of the screening charges and promote domain decay. The diffusion current value is independent of the number of scans and increases with rising temperature. The external current occurs during scanning, thus the charge decrease has to be proportional to the scan number. A similar behavior is also observed for the discrete scanning. Namely, the decrease of the diameter of the polarized areas created by discrete scanning in SBN61:Ce (Fig. 7) is a manifestation of the backswitching effect. The decrease of the threshold field needed for domain backswitching at elevated temperatures leads to a more pronounced effect of size reduction of the polarized area.

On the other hand, the decreasing piezoresponse on heating manifests the decreasing polarization on approaching the transition temperature. The temperature dependence of the relative piezoresponse (Figs 5a and 7b) can be well described by the critical temperature dependence similar as for the spontaneous polarization

1 $$P(T)=A{({T}_{cr}-T)}^{1/2}$$

The extracted value of the critical temperature *T*_*cr*_ = 67 °C (SBN61:Ce) is close to the value of the freezing temperature *T*_*f*_ measured in this crystal by analysis of the temperature dependence of the dielectric permittivity^28^.

It should be mentioned, that the screening charge decay caused by electronic diffusion and external current occurs at any temperature below as well as above the freezing temperature. Nevertheless, we observed that isothermal depolarization is strongly accelerated above the transition temperature.

In all studied samples, the poled state can be created both above and below the freezing temperature, but relaxation of the induced piezoresponse shows qualitatively different behavior in both cases. Typically, in relaxors the freezing temperature is associated with the loss of stability of the poled state. Indeed, for SBN61 (*T*_*f*_ ≈ 65 °C^12^) the piezoresponse contrast induced at room temperature decays to approximately 50% of its initial value in 2 hours and remains stable hereafter (compare Figs 3b and 4). The same happens at elevated temperatures, but below *T*_*f*_ (Fig. 5b). Above *T*_*f*_ *,* the induced response continuously decays down to zero (Fig. 5b). Also for SBN75 (*T*_*f*_ ≈ 20 °C^16^) the induced domain decays relatively fast, the piezoresponse contrast disappeared after 1 hour already (Fig. 3b). It is possible to attribute the observed change of the slope at 45 °C for SBN75 (Fig. 3a) to a crossover from the primarily backswitching driven decay of the induced domain to the depolarization due to instability of the induced ferroelectric state.

## Conclusions

The stability of domains written by a PFM tip in uniaxial SBN single crystals has been investigated in a broad temperature range. Above the transition temperature, the induced domains completely decay after a certain time. Below the transition temperature, only partial backswitching of the domains has been observed. The backswitching is promoted by the incompletely screened depolarization field. Diffusion of the screening charges upon heating results in further decay of the domains. The stability of the domains strongly depends on the scanning conditions. The grounded PFM tip acts as a collector of screening charges, which results in accelerated backswitching by repeated (continuous) scanning.

## Methods

The studied samples are plates of pure and Ce-doped (0.004 wt.% CeO_2_) strontium barium niobate single crystals Sr_*x*_Ba_1−*x*_Nb_2_O_6_ (SBN100*x*, SBN100*x*:Ce) with *x* = 0.61 and 0.75 grown by the modified Stepanov technique^29^ in Prokhorov General Physics Institute of Russian Academy of Sciences (Moscow, Russia) and by Czochralski method^30^ in University of Silesia (Katowice, Poland). The 0.6-mm-thick plates were cut normal to the polar axis and carefully polished.

Local poling was done at different temperatures ranging from 20 °C up to 90 °C (the last temperature is above the freezing temperature for all studied SBN compositions). PFM imaging was done using an *ac* voltage with amplitude *U*_*ac*_ = 3–10 V and frequency *f*_*ac*_ = 5–17.4 kHz applied between the conductive tip and the bottom electrode^31^.

Two atomic force microscopes were used: (1) MFP-3D (Asylum Research, USA) equipped with Ti/Ir-coated silicon tip-cantilever assemblies ASYELEC-02 (the spring constant is about 42 N/m, the resonant frequency is about 285 kHz, and the tip apex radius is about 25 nm) and (2) Probe NanoLaboratory NTEGRA Aura (NT-MDT, Russia) equipped with silicon DCP20 (the spring constant is about 48 N/m, the resonant frequency is about 420 kHz, and the tip apex radius is about 70 nm) and DCP11 (the spring constant is about 12 N/m, the resonant frequency is about 260 kHz, and the tip apex radius is about 70 nm) cantilevers (NT-MDT, Russia) having diamond-like conductive coating. The experimental data were processed using software packages WSxM (Nanotes, Spain)^32^ and Origin 8.6.

## Electronic supplementary material

Supplementary Information for Temperature Effect on the Stability of the Polarized State Created by Local Electric Fields in Strontium Barium Niobate Single Crystals
