# Supplementary material for: Temperature Effect on the Stability of the Polarized State Created by Local Electric Fields in Strontium Barium Niobate Single Crystals
Source: Sci Rep. 2017 Mar 9;7:125. doi: 10.1038/s41598-017-00172-1 (PMC5427817; doi:10.1038/s41598-017-00172-1)
Supplement: Supplementary file 1 — Supplementary Information for Temperature Effect on the Stability of the Polarized State Created by Local Electric Fields in Strontium Barium Niobate Single Crystals [file 41598_2017_172_MOESM1_ESM.pdf]

Supplementary Information for

Temperature Effect on the Stability of the Polarized State Created by Local Electric Fields in Strontium Barium Niobate Single Crystals

V. Ya. Shur[1], V. A. Shikhova[1], D. O. Alikin,[1] V.A. Lebedev[2], L. I. Ivleva[3], J. Dec[4], D. C. Lupascu[5], V. V. Shvartsman[5]

[1] Institute of Natural Sciences, Ural Federal University, 620000, Ekaterinburg, 51 Lenin Ave., Russia

[2] Faculty of Materials Science, Lomonosov Moscow State University, 119991, Moscow, GSP-1, 1-73 Leninskiye Gory, Russia

[3] Prokhorov General Physics Institute, Russian Academy of Sciences, 119991, Moscow, 38 Vavilova str., Russia

[4] Institute of Materials Science, University of Silesia, 40-007, Katowice, 4 Uniwersytecka str., Poland

[5]Institute for Materials Science and Center for Nanointegration Duisburg-Essen (CENIDE), University of Duisburg-Essen, 45141 Essen, Universitätsstraße15, Germany

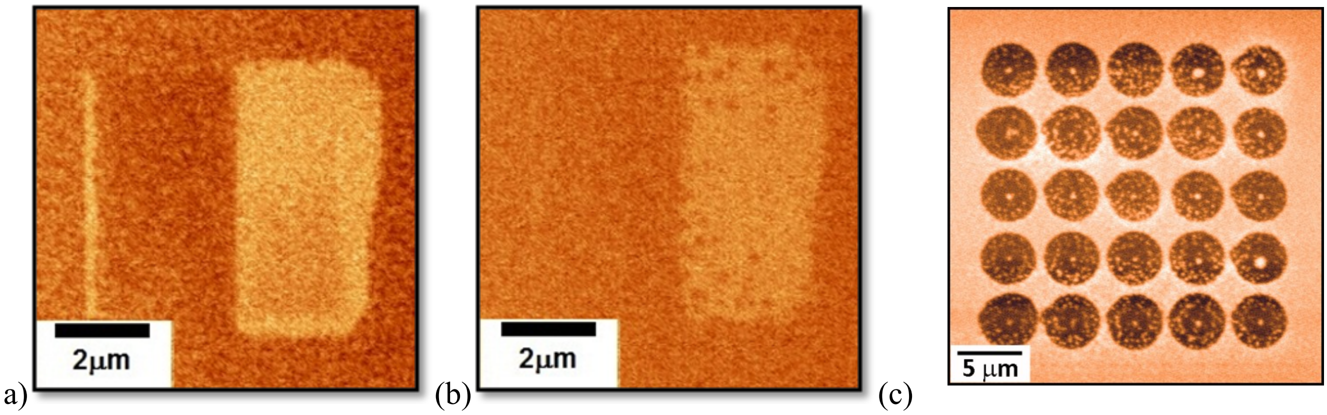

Figure S1. (a,b) PFM image of the recorded polarized area after "continuous" scanning in SBN61 at room temperature at (a) 0 min and (b) 120 min. U = 50V. (c) PFM images of domains created by "discrete" scanning in SBN61:Ce at room temperature, U = 200 V, pulse duration 1 s.
